# Seasonal changes in temperate woody plant phloem anatomy and physiology: implications for long-distance transport

**DOI:** 10.1093/aobpla/plab028

**Published:** 2021-05-25

**Authors:** Dustin M Ray, Jessica A Savage

**Affiliations:** Department of Biology, University of Minnesota Duluth, Duluth, MN 55811, USA

**Keywords:** Carbon transport, dormancy, phenology, sap viscosity, winter

## Abstract

Seasonal changes in climate are accompanied by shifts in carbon allocation and phenological changes in woody angiosperms, the timing of which can have broad implications for species distributions, interactions and ecosystem processes. During critical transitions from autumn to winter and winter to spring, physiological and anatomical changes within the phloem could impose a physical limit on the ability of woody angiosperms to transport carbon and signals. There is a paucity of the literature that addresses tree (floral or foliar) phenology, seasonal phloem anatomy and seasonal phloem physiology together, so our knowledge of how carbon transport could fluctuate seasonally, especially in temperate climates is limited. We review phloem phenology focussing on how sieve element anatomy and phloem sap flow could affect carbon availability throughout the year with a focus on winter. To investigate whether flow is possible in the winter, we construct a simple model of phloem sap flow and investigate how changes to the sap concentration, pressure gradient and sieve plate pores could influence flow during the winter. Our model suggests that phloem transport in some species could occur year-round, even in winter, but current methods for measuring all the parameters surrounding phloem sap flow make it difficult to test this hypothesis. We highlight outstanding questions that remain about phloem functionality in the winter and emphasize the need for new methods to address gaps in our knowledge about phloem function.

## Teaser

How does sugar movement change seasonally and can sugars be moved during the winter when temperate trees are largely dormant? We review the current knowledge of seasonal changes to the sugar-conducting tissue, the phloem, and use a simple model to facilitate a discussion of whether or not the phloem could be more active than previously assumed in some species.

## Introduction

**Figure 1.**
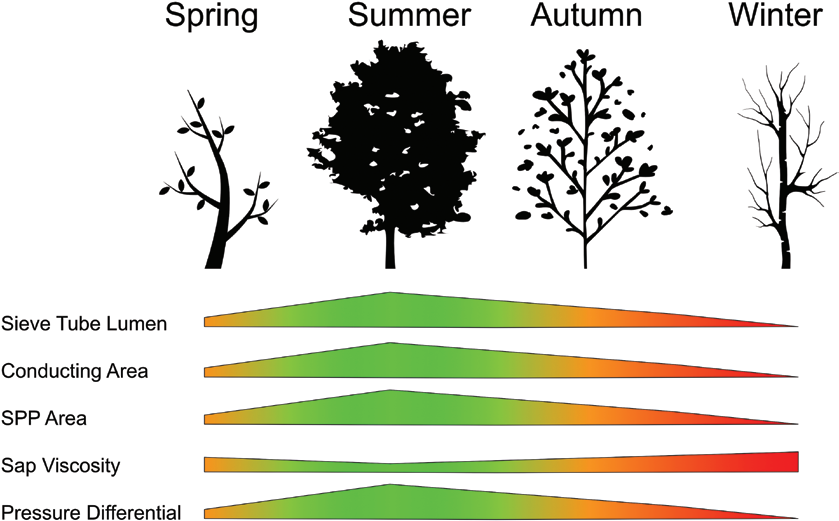
The effects of changes in anatomical and physiological parameters on phloem sap flow during the year. Polygon width indicates the relative change to the parameter listed in the left column and colours indicate relative effect on sap flow where orange is a moderate flow, green is optimal flow and red is little to no flow. SPP = Sieve plate pore.

In temperate environments, perennial plants exhibit cyclical shifts in resource allocation from the growth of flowers and leaves in the spring to leaf senescence and carbon sequestration during fall and early winter (Brüggemann *et al.* 2011; Palacio *et al.* 2018). These large changes in allocation require remobilization of carbon, water and nutrients in the vascular system and should be influenced by seasonal changes in vascular function (Fig. 1; Lechowicz 1984; Savage 2021). The phloem is particularly important during these periods of seasonal change because it transports carbon, nutrients and signalling molecules such as hormones, small RNAs and electrolytes (Thompson and Schulz 1999; Ruiz-Medrano *et al.* 2001; Kehr and Buhtz 2008) that can influence phenology directly (e.g. trigger senescence and flowering) or indirectly (e.g. impact circadian rhythm and apical dominance; Chincinska *et al.* 2013; Van den Ende 2014; Kim *et al.* 2018; Kim 2019, 2019; Kumar *et al.* 2019). Sugar itself can also serve as a signal for processes such as flowering (Horacio and Martinez-Noel 2013; Li 2016; Cho *et al.* 2018; Kim 2019). Therefore, understanding the capacity for signal and carbon movement by the phloem during the winter-spring transition is vital to being able to understand spring growth. Considering that there are limited data documenting seasonal anatomical changes to phloem physiology (Savage 2020), the extent that phloem transport function is reduced or compromised in the winter for many species is unclear.

Historically, seasonal studies of anatomy have been used to infer changes in phloem functionality (for a review see Evert 2006). Many studies have shown that while the classical view presented in even some recent plant anatomy textbooks is that carbon transport stops in the winter (e.g. Crang *et al.* 2018), anatomical studies suggest that there may be variation in species ability to transport carbon throughout the winter months (Evert 2006). Some species retain completely open and potentially active sugar-conducting conduits (sieve tubes; Tucker and Evert 1969), while others retain a reduced number of open sieve tubes or seal their sieve tubes with the polysaccharide callose either for the winter or permanently (Zamski and Zimmermann 1979; Fisher 1983; Evert 2006). In the late winter or early spring, new phloem tissue is produced by the cambium before xylogenesis (Tucker and Evert 1969; Lavrič *et al.* 2017) and in some species there are open sieve tubes adjacent to the cambium before division begins (Davis and Evert 1970; Aloni and Peterson 1997). In general, phloem development and re-activation is prioritized over xylem development in the early spring and new phloem in the stem is always produced before leaf-out (for review of vascular phenology, Savage and Chiune 2021).

Despite the prevalent assumption that sieve elements are often closed in the winter, there could be benefits to retaining some phloem function all winter to allow for transport of signals and resources throughout the plant body. For example, the phloem could support changes in carbon allocation leading to starch accumulation in floral buds as seen in *Prunus* and *Olea* during the winter (De la Rosa and Rallo 2000; Fadón *et al.* 2018). It could also support other types of growth including development of leaf primordia which continue to grow slowly throughout the winter in some species (Gordon *et al.* 2006). Overall, the greatest demand on the phloem is during peak growth in the early spring. During this time, the size of floral tissue in buds can increase four 100-fold (Savage 2019). Phloem transport capacity in the spring may be especially important for seasonally precocious species that undergo floral bud burst during the persistent threat of winter frost before there are leaves on the plant.

There is limited research that has attempted to directly link phloem anatomy with phloem function in the spring (Aloni and Peterson 1991) partly because the methods necessary to do so are limited. There has been a renewed interest in developing and optimizing methods for studying *in situ* phloem transport (Savage *et al.* 2013; Knoblauch *et al.* 2015; Ray and Savage 2020). Recent efforts have improved our ability to overcome problems with wounding and manipulation (Knoblauch *et al.* 2014; Howell *et al.* 2020), but studying the phloem during winter in temperate climates presents an added challenge because most trees do not have leaves and the source-sink dynamics utilized by many phloem physiological methods are reduced.

In this review, we consider what is known about how changes in phloem anatomy and plant physiology could impact transport of carbon throughout a plant in the winter and its potential implications for spring growth. Transport in sieve tubes is driven by a pressure differential created by local osmotic gradients in source and sink tissues (Münch 1930). We will consider how linear sap velocity (*ν*) is influenced by several key components: the pressure gradient (Δ*p*), sieve tube lumen area (*A*) and the tube resistance (*R*):


$$\begin{array}{l} \nu =\frac{\Delta p}{RA} \end{array}$$ (1)

We will discuss the ramifications of changes to hydraulic resistance and the source-sink differential, highlighting how the composition of the phloem sap, sieve element structure and sap viscosity could change seasonally with a focus on the winter. Our goal is to demonstrate that carbon transport in the winter is likely dynamic and there may be species-specific differences that could have implications for spring growth. Our analysis includes an in-depth discussion of the literature and a consideration of how methodology has shaped our perception of phloem physiology.

## Pressure Gradient

Movement of osmolytes into and out of the sieve tubes can occur either by active transport in the apoplast, or by active or passive transport in the symplast (Gamalei 1989; Lalonde *et al.* 2003; Rennie and Turgeon 2009). As osmolytes are loaded into sieve elements (the individual cells that make up the sieve tubes), high osmolyte concentration or low water potential draws water in, creating positive pressure to push the phloem sap to areas of low pressure where osmolytes are unloaded (sinks; Münch 1930). Because the pressure gradient that drives phloem sap flow is influenced by local processes in the source and sink tissue, understanding seasonal changes in phloem transport requires knowledge about source and sink activity, osmolyte concentration and composition, membrane permeability and enzyme activity in the phloem and adjacent cells.

### Source and sink activity

Studies of photosynthesis, growth, non-structural carbohydrate allocation and enzyme activity have shown dynamic shifts in source and sink activity during and between seasons (Gruber *et al.* 2013; Jyske and Hölttä 2015). In the fall, low photosynthetic rates can slow carbon transport by decreasing the sugars available for loading into the phloem sap (Ho 1976), which could impact pressure in the source tissue. However, phloem pressure does not have to change directly in proportion to changes in carbon fixation. In leaves after carbon is fixed, sucrose must be synthesized before sugars are loaded into sieve tubes. Anything that impacts the rate of carbohydrate synthesis could decouple photosynthetic rates and phloem transport. For example, at low temperatures, the activity of sucrose phosphate synthase, the enzyme that catalyses sucrose synthase, increases resulting in higher sucrose concentrations in *Spinacia* leaves (Guy *et al.* 1992). Similar complications may arise in the winter in species that can fix carbon when conditions become more favourable but are unlikely to matter once deciduous species become endodormant (Ogren 1997).

If a pressure gradient is maintained in the winter, it needs to be supported by different sources and sinks than during the growing season. Potential sinks that can drive flow include respiration, seed or fruit filling and leaf or flower growth which have different phenologies (Patrick 1997). In winter, developing flowers or leaves and respiration are the most likely active sinks but they should be weak. Stem respiration rates are greatly reduced in the winter (Ceschia *et al.* 2002; Damesin 2003), while leaf and floral buds appear to be varied in their winter growth and development. Bud growth often occurs in the final dormancy stage, ecodormancy, thus buds are more likely to be a sink near the end of winter (Faust *et al.* 1997; Horvath *et al.* 2003). As previously mentioned, some species appear to accumulate starch during the winter (De la Rosa and Rallo 2000; Fadón *et al.* 2018), creating a potential sink, but whether the carbon required for this process is supplied locally or transported in the vascular system remains unclear. It seems likely that sink strength decreases considerably in early winter and begins to increase in late winter as metabolism and growth begin in the buds.

Throughout the year, the relative proportion of sugars in the phloem sap fluctuates as carbon is allocated between different sources and sinks (Fisher 1983; Gruber *et al.* 2013; Jyske and Hölttä 2015). Phloem sugar concentrations within an individual tree may even vary when sampled at different heights during the same season (Pate 1998), suggesting that the winter dynamics of sugar transport may also vary by sampling location within an individual. The primary cause of changes in phloem sap composition during spring is the conversion of starch into soluble sugars through re-mobilization of stored carbon in stems, trunks and roots. In autumn and winter, phloem sap sugar concentration sampled by stylectomy increased to concentrations as high as double that of the spring and summer (Fisher 1983).

### Osmolyte composition

Species that produce flowers or leaves early in spring before freezing danger has passed need mechanisms to protect them from damage (Lineberger and Steponkus 1980;Peters and Keller 2009; Charrier *et al.* 2013) and the composition of the phloem sap may assist in the cryoprotection of sieve tubes. The contents of the phloem sap are complex, consisting of sugars, amino acids, proteins and small RNAs. Sugars (sucrose, glucose, fructose, mannose, galactose and raffinose) are present in the highest concentrations and likely confer some freezing tolerance (Dinant *et al.* 2010; Hijaz and Killiny 2014; Hijaz *et al.* 2016). For example, sucrose typically makes up the largest fraction of the phloem sap, and could provide cryoprotection for some species. The freezing point depression of 17 % sucrose (w/v), the optimal sucrose concentration for flow within the phloem (Jensen *et al.* 2013), is ~1.1 °C (Lide 2009). If the phloem sap sugar concentration were doubled, the freezing point would be depressed 3.37 °C (Lide 2009), possibly explaining the phloem transport range for *Salix viminalis* (−4 °C; Weatherley and Watson 1969). Many species can transport carbon at lower temperatures (*Salix exigua* −13 °C; Fisher 1983) indicating that there must be other forms of cryprotection in the phloem. Sieve elements can supercool when plunged in liquid nitrogen, though the process of supercooling appears to be reliant on hardening at cool temperatures prior to the onset of winter (Froelich *et al.* 2011). It is often assumed that if ice forms inside sieve tubes it is lethal, but it is also possible that there could be the formation of amorphous, uncrystallized ice allowing for vitrification of the tissue in some species (Hirsh *et al.* 1985; Debenedetti 1996).

Raffinose is another phloem osmolyte that confers osmoprotection (dos Santos *et al.* 2011), cryoprotection (Bachmann *et al.* 1994; Stushnoff *et al.* 1998; Pennycooke *et al.* 2003; Peters and Keller 2009), and inhibits the tendency of sucrose solutions to crystalize at low temperatures (Caffrey *et al.* 1988; Lipavská *et al.* 2000). The proportion of raffinose to sucrose in the phloem increases in autumn (Bachmann *et al.* 1994) and under cold conditions (Hinesley *et al.* 1992) and in frost resistant species (Parker 1963; Sakai and Larcher 1987; Lintunen *et al.* 2018), in response to changes in photoperiod and temperature (Wiemken and Ineichen 1993). The ratio of raffinose to other phloem sugars is highest at colder latitudes in both angiosperms and gymnosperms in Europe (Lintunen *et al.* 2018). Raffinose is present in minor leaf veins (Haritatos *et al.* 2000) of polymer-trapping species (Rennie and Turgeon 2009), some apoplastic-loading species, conifers (Hinesley *et al.* 1992), and can be made from sucrose as a response to abiotic stress (ElSayed *et al.* 2014). It is possible raffinose could also be made from sucrose within the phloem sap rather than being transported into the sieve tubes. It is not clear how the presence of raffinose affects the viscosity of the phloem sap at low temperatures, but modelling suggests that raffinose and similar sugars found in the phloem sap are more favourable to viscosity than sucrose (Lang 1978). Also, sucrose and raffinose solutions in glycine of the same concentration have similar viscosities (Ali *et al.* 2019).

Given its cryoprotective effects, raffinose could be a major contributor to the pressure gradient during temperate winters, as it appears to allow the phloem sap to remain at a viscosity conducive to flow longer than predicted for a simple sucrose solution. Raffinose has more carbon than sucrose, so fewer raffinose molecules are made for the same amount of carbon. If we assume a discrete level of carbon, having a greater proportion of raffinose in the phloem sap than sucrose would decrease its osmotic potential and therefore reduce source pressure. As a result, phloem sap flow could be altered as the temperature drops if sugar concentrations were modified in the source and sink sap. It is possible that during the winter genera with a portion of their sieve tubes open contain more raffinose in their phloem sap than species that completely occlude their sieve tubes, but we are not aware of any studies that have investigated the phloem sap contents and phloem overwintering strategies. Species that retain open and seemingly active sieve tubes in the winter should be studied to better understand the contents of the phloem sap and their contribution in sieve element cryoprotection.

## Hydraulic Resistance

Phloem hydraulic resistance within sieve tubes (*R*) can be affected by several variables that change seasonally including conduit anatomy and sap viscosity. Some of the resistance to flow in a sieve tube comes from the sieve element lumen (*R*_lumen_), which can be expressed as:


$$\begin{array}{l} R_{lumen}=\;\;\;\frac{8\eta L}{\pi r^{4}} \end{array}$$ (2)

where *η* is the sap viscosity, *L* is the path length from source to sink and *r* is the radius of the sieve tube lumen. Equation (2) demonstrates that anatomy (sieve tube radius) has a major effect on flow rate due to the fourth power relationship, i.e. narrower sieve tubes are significantly more resistant to flow than wider ones. Longer path lengths can also increase the resistance in this model, but sieve tube elements taper in diameter as a function of distance from base of the trunk to the tips of branches, which minimizes resistance conferred by conduit radius along the flow path (Petit and Crivellaro 2014; Liesche *et al.* 2016; Savage *et al.* 2017).

At least half of the resistance in sieve tubes is caused by the sieve plates, which occur at the ends of each sieve element (Savage *et al.* 2017; Stanfield *et al.* 2019). Jensen (2012), modified Equation (2) for a single sieve element, adding terms to account for the resistance of the sieve plate pore radii by using the mean pore diameter and standard deviation:


$$\begin{array}{l} R_{total}=\frac{8\eta L}{\pi r^{4}}+\frac{3\eta }{{r_{p}}^{3}}\;\frac{1}{N}\;\left[\frac{1}{1+3\beta^{2}}+\frac{\alpha }{1+6\beta^{2}+3\beta^{4}}\right] \end{array}$$ (3)

where *N* is the total number of pores in a sieve plate, *r*_*p*_ is the radius of the sieve plate, $\alpha =\frac{8l_{p}}{3\pi \bar{r_{p}}}$, and $\beta =\;\frac{\sigma }{\bar{r_{p}}}$, where $l_{p}$ is the length of the pore lumen, i.e. the plate thickness, $\bar{r_{p}}$ is the average pore radius and σ is the standard deviation. As such, the resistance for an entire sieve tube is the sum of the resistances for each sieve element ($R_{total}$) that are stacked to form a sieve tube.

### Sieve element anatomy and cross-sectional area

There are several ways that sieve element anatomy changes during the year. First, sieve elements produced in a single growing season are not always uniform in diameter. Elements produced during the fall are narrower and have a higher resistance to flow than those produced in the spring (Evert 2006; Prislan *et al.* 2013). It is not clear if this pattern is advantageous or is a result of developmental ties between the phloem and xylem (Jyske and Hölttä 2015; Ray and Jones 2018). Second, sieve plate resistance is increased when callose is deposited on the sieve plates (Mullendore *et al.* 2010; Jensen *et al.* 2012), a process that occurs seasonally in some species (Evert 2006). Plants with callose-occluded sieve plates either re-activate them by removing callose in the spring or differentiating new sieve tubes in the late winter and early spring. If callose only partially covers pores, it may reduce the sieve plate pore diameter, but still allow flow to occur. It is unclear if callose is present in sieve tubes under normal conditions because most anatomical studies of sieve tubes in woody trees use methods that cause mechanical damage eliciting a callose response and fix the collected tissue too slowly resulting in callose artefact (Esau and Cheadle 1961; Evert and Derr 1964). Clearly more work focussed on callose and its presence seasonally within the phloem is needed (Montwé *et al.* 2019).

The total phloem conducting area changes seasonally and likely varies by the ability to keep sieve elements open in the winter. In species that retain open sieve tubes throughout the year, transport capacity would be similar in the winter and summer months, whereas in species that occlude all sieve tubes during the winter, all flow would stop during winter. Those species that retain a small number of seemingly open sieve elements, however, would have less conducting area during the winter and likely a lower capacity for carbon transport in early spring (Savage 2020). Seasonal anatomical changes are likely coordinated with sink and source strength, ensuring flow when it is necessary. Smaller diameter sieve tubes produced at the end of the season may allow flow to continue in the winter by facilitating easier build-up of pressure to drive flow. Research specifically focussed on such changes is needed to better contextualize the seasonal changes of anatomy in the sieve tubes and its implications for the winter-spring transition.

### Changes in viscosity

Viscosity increases exponentially with concentration in sucrose solutions indicating that sap concentration could easily impact resistance to flow in sieve tubes (Morison 2002; Sevanto 2014). Considering the viscosity in sucrose solutions also increases at lower temperatures and with higher concentrations of solutes (Telis *et al.* 2007), viscosity should build up during the transition from fall into winter when phloem sap osmolyte concentrations increase (Fisher 1983). If this increase is high, viscosity could be a limiting factor for movement of signalling molecules as well as carbon in the winter, similar to what may theoretically occur in the phloem during drought (Sevanto *et al.* 2013; Sevanto 2014; Gaylord *et al.* 2015).

## The Effects of Winter on Phloem Sap Flow

To directly illustrate how seasonal changes in sieve tube anatomy, callose deposition and changes in source and sink activity impact phloem sap flow, we modelled phloem transport velocity using a simplified model and published values for *Quercus rubra* (Savage *et al.* 2017). We calculated theoretical flow rates for a single sieve tube within a 27 m tall individual assuming that the sieve tube is the length of the tree. We chose this species because all of the parameters required to calculate resistance, including the number of sieve elements per metre, sieve pore area and diameter and sap flow using a pipe-flow model have been previously measured (Münch 1930; Huber *et al.* 1937; Savage *et al.* 2017). Our model considers the viscosity of an aqueous sucrose solution within a single sieve tube and demonstrates the effects on flow that result from changes in sap viscosity, the pressure gradient, and sieve plate callose occlusion at a variety of temperatures.

**Figure 2.**
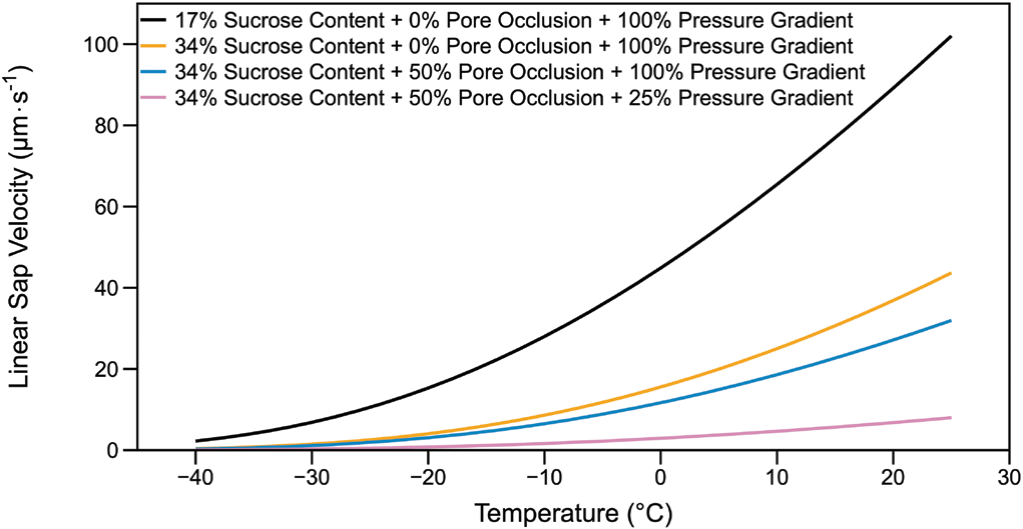
The theoretical effects of sucrose concentration, sieve plate pore occlusion and pressure gradient changes on linear sap flow in the phloem. Under optimal conditions, such as in the summer, phloem sap concentration is around 17 % sucrose, sieve plate pores are not occluded and the pressure gradient is unchanged (black line). Linear sap velocity is reduced if sucrose concentration is doubled as may happen in the winter (orange line). Further decreases in linear sap velocity occur if the sieve plate pores become partially occluded with callose (blue line) in the late fall or winter. The lowest linear sap velocities occur when the pressure differential is also reduced (25 % of normal) as may occur when leaves are not present during late fall and winter (purple line).

We calculated viscosities for an aqueous sucrose solution at a range of temperatures experienced by trees in temperate climates using the relation from Mathlouthi (1995), linear sap velocity using Equation (1) and resistance using Equation (3), which accounts for both the sieve plate pores and sieve element lumen. To account for the widening of sieve elements from the tree apex towards the base of the trunk (Savage *et al.* 2017), we divided our 27 m tall tree into three 9-m segments calculating the resistance first for a single sieve element within each segment and then for the sum of resistances for all the sieve elements within that segment. The sum of each segment was added together to calculate the resistance for an entire sieve tube as in Clerx (2020). Finally, to convert values from volumetric sap flow to the more commonly used linear sap velocity, we divided the volumetric sap velocity by the area of a circular sieve element of radius ~24 µm, the radius of a sieve tube at approximately the midpoint of the tree (see Equation (1)). This assumption ignores the widening of sieve elements from branch tip to tree base and represents only a single point measurement, though the maximal velocity in the model (~100 µm s^−1^; Fig. 2) is near what has been reported for *Q. rubra* (Huber *et al.* 1937).

We note that these equations, while used in many complex modelling studies (Jensen *et al.* 2013; Knoblauch *et al.* 2016; Savage *et al.* 2017; Clerx *et al.* 2020) are only applicable under specific conditions: they consider a single sieve tube that is continuous along the length of the tree that is loaded at the branch tip, widens from branch tip to tree base, and is unloaded at the trunk base. The resistance equation considers an empty pipe (no organelles) with no lateral water movement along the flow path. This model also assumes a sieve tube with impermeable walls, which may not be the case for the sieve tubes (Phillips and Dungan 1993; Sevanto 2014; Stanfield *et al.* 2017). While simple, this model allows us to illustrate the factors that impact flow within the phloem and understand the factors that may allow some species to undergo the transition to spring more quickly than others.

The sap concentration of *Q. rubra,* was reported as 17.1 % in the summer (Münch 1930; Jensen *et al.* 2013) and we used that as our base value for viscosity calculations (Fig. 2, black line). We considered the additive effect of three possible changes to the phloem in the winter and their combined effect on velocity. First, phloem viscosity likely increases due to lower temperatures and an increase in phloem sap sugar concentrations. In *Salix*, sugar content of the phloem sap doubles in the winter (Fisher 1983). If similar sap concentration changes occur in *Q. rubra*, the sucrose concentration would be near 34 % in the winter (Fig. 2 orange line). Next, compounding with the increase to the sap concentration, we added a reduction in the size of the sieve plate pores due to callose accumulation (Fig. 2 blue line). We used a 50 % reduction in the sieve plate pore radius of all sieve plate pores throughout the phloem but note that the degree to which the sieve plates become occluded in functional winter sieve elements during the winter is not documented. Finally, with the loss of leaves in autumn, the pressure gradient within sources and sinks is likely greatly altered. Here we used a 75 % reduction in the pressure gradient (Fig. 2 purple line), but again note that more seasonal studies are necessary to determine the pressure gradient within trees during the winter.

Our model suggests that sap flow within a sieve tube can continue below freezing if any one variable considered by the model is reduced in value, but the compounding effects to all variables appear to reduce the capacity for sap flow below freezing temperatures. At 0 °C, for example, the calculated velocity considering an increased sap concentration, partially occluded sieve plate pores, and a reduced pressure gradient is ~3 µm/s, when compared with ~45 µm/s at the same temperature with open sieve elements and a ‘normal’ pressure gradient within the sieve tube. Our model supports the dominance of the pressure gradient in limiting sap velocity (Fig. 2). Our model suggests flow is possible well below 0 °C if the pressure gradient is maintained. Thus, local flow could occur over shorter distances if a pressure gradient could be generated, for example within a portion of a single branch, rather than between the tips of the branches and base of the tree. Flow could also occur in regions of the plant body where wider diameter sieve tubes occur such as in a tree trunk, where sieve elements are wider when compared with distal stems (Savage *et al.* 2017; Clerx *et al.* 2020), especially if there are differences in early versus late sieve element diameter, a factor not included in our model.

Our model also suggests that in order to restore flow, even in temperatures below zero, trees need only to alter their sink activity. When put into context with the various overwintering strategies, this model suggests that retaining some open sieve tubes may allow species to quickly re-activate in the spring because flow could be restored by expanding leaves or flowers in buds acting as sinks. Species that must differentiate and mature new sieve tubes to resume phloem function in the spring may not be able to transport a high enough quantity of resources to support growth early in the spring. Alternatively, species could restart phloem flow if the xylem becomes a carbon sink. Sucrose may, for example, be moved into the xylem to reduce the likelihood of spring embolism within the xylem (Zwieniecki *et al.* 2015; Konrad *et al.* 2019).

It is likely that many different strategies are utilized to facilitate phloem sap flow in late winter and are currently unrecognized due to our limited knowledge of species-specific phloem overwintering and phenology. *Acer negundo,* for example, flowers precociously early in the season and does not appear to occlude or otherwise close sieve tubes in the winter (Tucker and Evert 1969), while *Populus tremuloides,* which also flowers precociously often before *A. negundo* appears to produce new phloem from overwintered, partially differentiated phloem ‘mother cells’ (Davis and Evert 1968). Broad seasonal studies that measure phloem flow while documenting phloem overwintering strategies and phenology are necessary to fully understand the advantages and disadvantages to open sieve elements, but those studies will require methods that allow for seasonally repeated measures on large numbers of species or individuals.

## Outstanding Questions about Phloem Seasonality

Before plants can invest in significant new growth in the spring, they need to have a partially functional vascular system, but the timing and mechanics of the phloem’s transition from winter to spring is still understudied. Our knowledge of seasonal changes to phloem physiology is limited to a small number of studies documenting phloem functional area, sieve tube pressure and sap flow change during the winter and spring. One reason for this gap in knowledge is long-recognized methodological challenges that arise from studying the phloem, a pressurized, membrane-bound system (Knoblauch and van Bel 1998). Further limitations in studying phloem seasonally arise from a high level of among-species-variation in seasonal responses and the need for time-series data. To determine whether seasonal changes in the phloem have implications for spring phenology, we need to answer three key questions, outlined below. With each question we consider challenges and potential future direction for expanding our knowledge in these areas.

### How much does sieve element conducting area change seasonally?

A critical part of understanding seasonal changes in flow is determining the area of conducting sieve tubes in the phloem. In our model, differences in the conducting area would influence the volumetric sap flow in the phloem (product of sap velocity and conducting area). While not explicitly explored in our model, the total conducting area directly affects the sap volume that can be conducted through the plant body. Current methods for identifying functional sieve tubes using TEM or light microscopy can be time consuming and complex to interpret (Knoblauch and Oparka 2012). More targetted approaches with a higher specificity to active sieve tubes are necessary to get enough replication of time-series data to examine seasonal changes in phloem transport area. Transgenic expression of fluorescent proteins specific to sieve tube walls is used in *Arabidopsis* (Yang and Russell 1990; Thompson and Wolniak 2008). This method allows for rapid assessment of sieve tube area but does not indicate whether conduits are functional and would, therefore, require a subsequent screening using aniline blue or immunogold staining and detection with TEM to confirm functionality (Prislan *et al.* 2013). The recent discovery of a monoclonal antibody, LM26, that binds to the epitope on a pectin specific to sieve tube cell walls of herbaceous crop species, also appears promising (Torode *et al.* 2018). This epitope appears to be present in the sieve tube walls of two species of *Populus* only at times when the sieve tubes have been previously described as actively conducting (Ray and Savage 2020). Immunostaining to detect the LM26 epitope is relatively straightforward and can be conducted on fresh or fixed tissue and completed in less than a day, which could allow for analysis of more samples and more accurate measurements of active sieve tubes. The LM26 antibody may become even more powerful if combined with Fourier Transformed Infrared Spectroscopy (FTIR), which involves the analysis of infrared absorption to identify individual chemical components of a sample. In plants, FTIR has been used to identify cell wall components in *Phaseolus* (Alonso-Simón *et al.* 2004) and was recently used to map the sucrose gradients of frozen cross-sections in cereal crops and *Arabidopsis* at resolutions of near 12 µm (Guendel *et al.* 2018). If applied to woody plants using plunge frozen microcores, FTIR spectroscopy could be used to discern the sugar composition of actively transporting sieve elements. Next steps should test the applicability of these techniques to larger samples sizes and a wider variety of species and growth forms. The FTIR work should also be replicated in conjunction with techniques that can identify active sieve elements, such as the sap flow measurements to confirm the results of the FTIR technique can provide reliable results specific to sieve tubes and to compare those results to those of other more established methods such as stylectomy. If such confirmational studies suggest that FTIR is able to discern the sugar contents within the sieve tubes, it could be used to analyse the seasonal change in the phloem sap sugar content across entire cross-sections.

### How does the phloem pressure gradient change seasonally?

Our model suggests that the pressure gradient in the phloem may play a critical role in regulating seasonal changes in sap flow. A large reduction in the pressure gradient during winter is likely for winter deciduous species, because the leaves are the primary source of sugar production most of the year. We assumed a 25 % reduction in the phloem pressure gradient, but the actual value is unknown. There is currently limited data on phloem pressure (Hammel 1968; Wright and Fisher 1980; Lee 1981; Knoblauch *et al.* 2016; Savage *et al.* 2017) and less research that relates to seasonality (except see Mencuccini *et al.* 2013). Sieve tube pressure has been measured with three techniques: sap-feeding insects using stylectomy, pressure sensors and more recently with pico gauges (Hammel 1968; Wright and Fisher 1980; Lee 1981; Knoblauch *et al*. 2014, 2016; Savage *et al.* 2017). Pico gauges are currently the most promising technique for directly measuring phloem turgor pressure and they work in some woody species (Knoblauch *et al.* 2016; Savage *et al.* 2017). More widespread use of these gauges could yield insights into the seasonal dynamics of sources and sinks, but they require very finely pulled pipettes and the ability to visualize their insertion into the sieve elements, making this technique difficult to implement at the sample sizes necessary for survey-level studies. Future studies should apply the pico gauge technique to more species and investigate the diurnal and seasonal trends of sieve tube turgor with a focus on winter. This could provide crucial empirical data about the location and strength of the sources and sinks during times when leaves are not present.

### How does the sap flow rate change seasonally?

The clearest way to understand seasonal changes in phloem function is to measure or visualize flow rate directly. Our model suggests that sap flow can continue below freezing temperatures only if the pressure gradient is able to be maintained and if the phloem sap concentration remains relatively close to that for optimal flow (17 %) rather than doubling as has been documented in *Salix* (Wright and Fisher 1980). The ideal approach to answer this question would be directly measuring sap flow during seasonal studies. Unfortunately, many common methods require highly specialized equipment (Magnetic Resonance Imaging, isotope studies, microCT) that in some cases must be customized to the specific individual being studied. While these techniques have provided essential information on phloem function (Windt *et al.* 2006; Homan *et al.* 2007; Devaux *et al.* 2009; Brüggemann *et al.* 2011; Brodersen and Roddy 2016), they are not amenable to the large sample numbers (and large sampling areas) required for measuring seasonal changes in phloem function in multiple species. Other methods that utilize phloem-mobile fluorophores may allow for greater replication, but currently exhibit variation in their species-specificity, which may be related to phloem loading type (Savage *et al.* 2013; Knoblauch *et al.* 2015). Because there is currently no technique that would allow for a wide-scale screening of winter sap flow across species, future work should focus on testing the validity of using different anatomical techniques to assess function. If future research could demonstrate a clear connection between seasonal changes in phloem function and anatomy, it would be possible to better judge differences in phloem function across species.

## Conclusions

Carbon and nutrients are mobilized in the phloem along with a host of signalling molecules that support the seasonal pulses of growth and senescence (van Bel *et al.* 2013). Though seasonal carbon allocation is well studied, anatomical and physiological changes to the phloem are not well documented. In this paper, we highlight the dynamic nature of phloem transport and the diversity of factors that could influence phloem transport in the winter and during the winter-spring transition from seasonal changes in source and sink activity to changes in sieve tube anatomy. We use a simple model to demonstrate the importance of phloem pressure in determining flow at low temperatures and consider the implications for different overwintering strategies for the timing of phloem re-activation in the spring. Future work should focus on gathering more time-series data on phloem anatomy and physiology and overcoming methodological barriers to collecting this type of data. In a time when plant phenology is changing in response to climate change, it is critical that we develop a more comprehensive perspective on seasonal changes in plant physiology and resource allocation.

## Data Availability

The annotated R code and related data used to make the phloem model can be accessed from DRYAD (doi:10.5061/dryad.rbnzs7hbg).

## Notes

**Plants, Ecosystems & Climate. Chief Editor:** Mary Heskel
